# Fc gamma RIIb expression levels in human liver sinusoidal endothelial cells during progression of non-alcoholic fatty liver disease

**DOI:** 10.1371/journal.pone.0211543

**Published:** 2019-01-29

**Authors:** Tomoko Ishikawa, Hiroshi Yokoyama, Tomokazu Matsuura, Yoko Fujiwara

**Affiliations:** 1 Institute for Human Life Innovation, Ochanomizu University, Bunkyo-ku, Tokyo, Japan; 2 Division of Gastroenterology and Hepatology, Department of Internal Medicine, The Jikei University School of Medicine, Minato-ku, Tokyo, Japan; 3 Department of Laboratory Medicine, The Jikei University School of Medicine, Minato-ku, Tokyo, Japan; 4 Graduate School of Humanities and Sciences, Ochanomizu University, Bunkyo-ku, Tokyo, Japan; The University of Hong Kong, HONG KONG

## Abstract

Liver sinusoidal endothelial cells (LSECs) play a pivotal role in hepatic function and homeostasis. LSEC dysfunction has been recognized to be closely involved in various liver diseases, including non-alcoholic steatohepatitis (NASH), but not much is known about the fate of the scavenger receptors in LSECs during NASH. Fc gamma receptor IIb (FcγRIIb), known as a scavenger receptor, contributes to receptor-mediated endocytosis and immune complexes clearance. In this study, to elucidate the fate of FcγRIIb in the progression of non-alcoholic fatty liver disease (NAFLD), we examined FcγRIIb levels in NAFLD biopsy specimens by immunohistochemistry, and investigated their correlation with the exacerbation of biological indexes and clinicopathological scores of NASH. The FcγRIIb expression levels indicated significant negative correlations with serum levels of blood lipids (triglyceride, total cholesterol, high-density lipoprotein-cholesterol), type 4 collagen and hyaluronic acid, which are involved in hepatic lipid metabolism disorder, fibrosis, and inflammation, respectively. However, there was no significant difference of FcγRIIb expression levels among the pathological grades of NAFLD. During NAFLD progression, inflammation and fibrosis may influence the expression of FcγRIIb and their scavenger functions to maintain hepatic homeostasis.

## Introduction

Non-alcoholic fatty liver disease (NAFLD) is the hepatic manifestation of metabolic syndrome and is categorized into non-alcoholic fatty liver (NAFL), with simple fatty liver, and non-alcoholic steatohepatitis (NASH), with inflammation and fibrosis. NAFL is a benign disease, but NASH can lead to severe chronic hepatic diseases, such as advanced fibrosis [1], cirrhosis [2], and hepatocellular carcinoma (HCC) [3, 4]. In 1998, a two-hit model [5] was proposed as a mechanism of NASH pathogenesis. The first hit is fat accumulation in the liver parenchyma caused by obesity and insulin resistance, and subsequent second hits are several cellular stresses including oxidative stress, mitochondrial dysfunction, and apoptosis stress. In recent years, a multiple parallel-hit hypothesis [6] has been attracting a lot of attention. Namely, the multiple factors (e.g., inflammation from gut-derived endotoxin or adipocytokines, endoplasmic reticulum stress, and innate immunity) may act in parallel and lead to the progression of steatosis, hepatocellular ballooning, inflammation, and fibrosis. There are many cases in which patients are not obese or diabeti, and they are asymptomatic until they reach a severe period of NAFLD. So it is necessary to elucidate the details of clinical phenotypes in the early stages of NASH.

Recent reports suggest that liver sinusoidal endothelial cells (LSECs) may play a pivotal role in the onset and progression of chronic hepatitis and liver cancer [7].　It is well known that the major functions of LSECs are [1] to control the transport of molecules in blood to the hepatocytes via fenestrae, and [2] to remove foreign or unwanted materials by means of scavenger receptors.

LSECs are the most permeable endothelial cells due to the presence of fenestrae and lack of basement membranes, and play a fundamental role in maintaining hepatic homeostasis. Therefore, the loss of these specific structural features is responsible for causing dysfunction of LSECs, and disrupting hepatic homeostasis [8].　Capillarization, characterized by the loss of fenestrae, the formation of tight junctions, and the expression of vascular endothelium-associated markers CD31, CD34, and von Willebrand factor in LSECs, occurs in human cirrhosis and chronic hepatitis [9], rat chronic liver disease induced by thioacetamide administration [10], and several pathological models *in vivo* and *in vitro* [7]. Capillarization of LSECs plays a key role even in the onset and progression of NAFLD/NASH [11]. Capillarization and sinusoidal communication are implicated in hepatic fibrosis, and the protection of LSECs may be an effective strategy to avoid fibrosis initiation and progression [10, 12]. The other noteworthy role of LSECs is the clearance function by means of scavenger receptor (SR) from the peripheral blood. The term “scavenger receptor” was originally coined to denote a cell surface receptor on the macrophage that mediates endocytosis and degradation of waste materials [13]. Thereafter, however, the expression of SRs has been reported on several cell types. PrabhuDas M et al. proposed the nomenclature and classification of SRs into 10 classes termed SR-A to SR-H [14]. The LSECs express SR-A (macrophage SR), SR-B (CD36), and SR-H (stabilin-1, stabilin-2), but do not express SR-D, SR-E, SR-F, and SR-G [15]. In physiological conditions, LSECs express three types of receptors that mediate the endocytosis of waste materials, hyaluronan receptors (stabilin-2, lymphatic vessel endothelial hyaluronan receptor-1 (LYVE-1)), mannose receptors (CD206), and a low-affinity gamma immunoglobulin Fc region receptor IIb (FcγRIIb) [15–17].

Both stabilin-1 and stabilin-2, which are hepatic hyaluronan clearance receptors, have been purified from rat livers and were characterized by Polits et al. [18]. Serum hyaluronan is a recognized non-invasive marker of liver fibrosis, and can accurately and independently predict mortality in patients with liver disease [19, 20]. Stabilin-1 and stabilin-2 are involved in the LSEC endocytosis of oxidized low-density lipoprotein (oxLDL), but not in the Kupffer cell endocytosis via the clathrin-mediated pathway [21]. In the human HCC and a murine HCC model, loss of stabilin-2, LYVE-1, and FcγRIIb in LSECs is noted in the majority of liver tumours [22].

The FcγRIIb is the sole inhibitory FcγR, which contains an immunoreceptor tyrosine based inhibitory motif (ITIM) in its cytoplasmic domain [23, 24]. FcγRIIb is mainly present on the surface of all leukocytes except NK cells and T cells, and is involved in regulating immunoreactivity. FcγRIIb is also expressed in hepatic sinusoids [25, 26]. FcγRIIb contributes to the clearance of small immune complexes in hepatic sinusoids [27].　Moreover, the expression of FcγRIIb in endothelial cells has been suggested to be associated with hepatic and cardiovascular diseases [28, 29]. As a ligand for FcγRIIb, fibrinogen-like protein 2 (FGL2), which is known to have immunomodulatory function, has been reported to have an affinity for FcγRIIb [30]. Interestingly, the plasma level of FGL2 is higher in patients with NAFLD [31] and liver cirrhosis [32].

These scavenger receptors on LSECs share the clearance task, and their downregulation may lead to increased blood concentrations of their specific ligands: hyaluronan, oxLDL, type IV collagen (IVCol), IgG and perhaps FGL2. However, not much is known about the fate of the endocytic receptors during human NAFLD. In the present study, we aimed to elucidate the correlations of FcγRIIb expression levels in LSECs of NAFL and NASH patients with clinical biochemical and pathological phenotypes. Furthermore, we examined another scavenger receptor, stabilin-2 expression, in LSECs of NAFLD patients.

## Materials and methods

### Patients

We analyzed 26 patients with NAFLD who had undergone liver biopsy for definitive diagnosis at Jikei University Hospital between 2011 and 2015 and were histologically diagnosed with NAFL or NASH. Fasting blood samples were collected from patients before liver biopsy. Patients who had been clinically diagnosed with other liver diseases or had consumed more than 20 g of alcohol per day were excluded from this study. Two patients with hepatitis B surface antigen (HBsAg) positive were included. This study was carried out with the approval of the Ethics Committee of the Jikei University School of Medicine, and the Ethics Committee of Ochanomizu University. Informed consent was obtained from all patients.

### Routine histological and biochemical analysis

For routine histology, liver biopsy samples were fixed with 4% paraformaldehyde in phosphate-buffered saline (PBS) and embedded in paraffin blocks. Sections, 3 μm thick, were stained with hematoxylin-eosin (H-E) and Masson’s trichrome for routine examination. The pathological investigation was performed by a board-certified pathologist of the Japanese Society of Pathology, and the histological grade of steatosis, inflammation, ballooning, fibrosis stage and NAFLD activity score (NAS) were determined using NASH Clinical Research Network criteria proposed by Kleiner et al [33].

Basic and diagnostic biochemical tests were obtained by routine methods. In this study, we analyzed the number of blood platelets (Ptl), serum levels of aspartate aminotransferase (AST), alanine aminotransferase (ALT), alkaline phosphatase (ALP), gamma-glutamyl transpeptidase (γ-GTP), total bilirubin (TB), total protein (TP), albumin (Alb), triglyceride (TG), total cholesterol (TC), low-density lipoprotein-cholesterol (LDL-C), high-density lipoprotein-cholesterol (HDL-C), fasting blood sugar (FBS), hemoglobin A1c (HbA1c), VICol, and hyaluronic acid (Hyal).

### Immunohistochemistry and morphometry

Immunohistochemical staining of FcγRIIb was performed on liver biopsy samples. The paraffin sections were deparaffinized and treated with 10 mM citric buffer (pH6.0) for 10 min at 95°C for antigen unmasking. Endogenous peroxidase activity was exhausted by 3% hydrogen peroxide. The sections were then pre-incubated with 5% normal goat serum-1%BSA in PBS, and incubated with a primary rabbit anti-human FcγRIIb antiserum (Peptide Institute, Osaka, JAPAN) or a rabbit anti-human stabilin 2 antiserum (abcam). Secondary antiserum from Histo Fine simple stain (MAX-PO Multi, Nichirei, Tokyo, JAPAN) was used, and detected with diaminobenzidine (DAB) and then counterstained with hematoxylin. The FcγRIIb expression level was morphometrically quantified on microphotographs taken with a 20 x objective lens on a BZ-X710 microscope and Hybrid Cell Count software (KEYENCE, Tokyo, JAPAN) as follows. For all specimens, as much fields as possible of the hepatic parenchymal area was photographed (12–35 fields per patient). The procedures to extract the whole tissue area and FcγRIIb positive area were determined, respectively, as shown in S1 Fig. Then the areas were automatically measured under the same conditions. To avoid observer-related bias, morphological observation and evaluation were blindly performed, i.e. the observer was unaware of the diagnosis and pathological scores.

### Statistical analyses

Statistical analyses were performed using SPSS Statistics 23 software (IBM, NY, USA). Comparisons of FcγRIIb expression levels with sex, diagnosis, and clinicopathological scores were performed by the Mann-Whitney or the Kruskal-Wallis nonparametric test. A P-value <0.05 was considered to be significant. Investigation of associations between FcγRIIb expression and body mass index (BMI) or serum biochemical values were performed by the Pearson correlation coefficient test. R-values(±) >0.7, >0.4, >0.3 were considered to have a strong, medium, and small correlation, respectively.

## Results

### Patient characteristics

Table 1 summarized the age, sex, BMI, and pathological characteristics of the 26 patients analyzed in this study. The number of patients was 13 for both males and females, and the mean ages for males and females were 48.5 and 56.6 years, respectively. With the biopsy-proven pathological examination, two patients were diagnosed as NAFL and 24 patients as NASH. In Table 1, the two patients diagnosed with NAFL are listed from the top, followed by the 24 diagnosed as NASH, in ascending order of NAS and then ascending ALT value. This suggests that the serum ALT level is not a sufficient indicator of NASH progression, though it is used as one of the screening criterions for NAFLD.

**Table 1:** The patient characteristics.

Diagnosis	Age	Sex	BMI	Blood Test																Pathological Stage				
				AST	ALT	ALP	γ-GTP	TB	TP	Alb	Plt	TG	TC	LDL-C	HDL-C	FBS	HbA1c	ⅣCol	Hyal	Steatosis	Inflammation	Ballooning	Fibrosis	NAS
				(U / L)	(U / L)	(U / L)	(U / L)	(mg / dL)	(g / dL)	(g / dL)	(10^4^ / μL)	(mg / dL)	(mg / dL)	(mg / dL)	(mg / dL)	(mg / dL)	(%)	(ng / mL)	(ng / mL)					
NAFL	54	F	23.6	21	14	334	30	0.4	7.0	3.8	530	197	227	160	67	103	5.8	117	<10	1	1	0	0	2
NAFL	43	F	31.3	78	141	249	663	0.8	6.1	3.5	89	316	304	192	112		4.7			3	0	0	0	3
NASH	73	M	23.0	46	50	265	40	0.9	7.3	4.1	220	98	209	153	56		6.0	127	83	1	0	1	1B	2
NASH	54	M	28.4	33	64	306	51	0.8	7.5	4.6	257	241	216	183	33	101	5.8	115	38	2	0	0	1A	2
NASH	66	F	30.2	43	23	318	18	0.6	6.9	3.5	247	185	163	120	43	117	5.8	162		1	1	1	2	3
NASH	65	F	23.0	34	148	440	1871	0.5	6.1	3.7	415	158	293	170	123	139	8.6			1	1	1	1B	3
NASH	46	M		32	54	150	27	0.9	6.9	4.2	269	116	192	137	55		5.8		23	3	1	0	1A	4
NASH	33	F	18.4	140	179	213	30	0.7	8.6	4.9	362	709	355	218	137	161	7.6	148	25	1	1	2	1A	4
NASH	38	M		89	205	147	109	1.6	6.0	4.2	163	252	174	149	25	301	10.6	86	44	2	1	1	3	4
NASH	58	F	22.4	170	206	220	70	0.7	8.0	4.9	225	111	179	130	49	114	5.7	196	64	2	1	1	3	4
NASH	34	M	34.6	153	259	241	352	1.6	7.0	4.5	171	206	204	167	37	214	8.9	149	45	2	1	1	3	4
NASH	65	F	30.0	34	17	243	35	0.6	7.4	2.8	199	78	155	94	61	93	5.6	320		1	2	2	3	5
NASH	57	M	25.3	25	32	382	34	0.4	7.3	3.8	77	108	168	102	66	138	6.4	218	203	1	2	2	3	5
NASH	53	F	29.0	40	50	391	28	0.8	7.3	3.9	230	152	180	144	36	100	6.7	147	258	1	2	2	3	5
NASH	58	M	26.4	64	61	344	106	0.7	7.6	4.5	207	196	212	148	64	100	5.4			1	2	2	3	5
NASH	68	F	31.6	60	78	407	26	0.6	7.4	4.0	119	126	172	93	54	100	6.1	151	78	1	2	2	3	5
NASH	46	M	30.5	59	93	202	182	0.8	7.2	4.6	209	216	197	98	99	126	6.7			2	2	1	1B	5
NASH	47	M	28.7	88	156	372	49	1.2	7.8	4.9	186	148	262	181	81	104	5.6	124	25	3	1	1	1A	5
NASH	47	M	36.7	148	183	411	86	1.4	7.6	4.4	196	205	215	174	41	117	6.1	206	20	2	2	1	3	5
NASH	31	M	29.0	221	267	191	119	1.0	7.8	5.0	163	364	249	176	73	145	6.4	297	72	2	1	2	1B	5
NASH	36	M	36.9	181	327	231	104	1.6	7.3	4.6	263	134	180	141	39		7.1	167	24	3	1	1	2	5
NASH	67	F	23.4	73	51	209	18	0.7	8.3	4.4	235	76	162	117	45	90	6.1	176		2	2	2	3	6
NASH	50	F		37	66	211	29	1.0	7.9	4.3	215	163	185	147	38	101	5.8	113	14	2	2	2	1B	6
NASH	64	M	25.8	67	77	242	47	1.2	7.0	3.8	165	431	124	92	32	123	5.9	162	26	2	2	2	3	6
NASH	60	F	23.7	70	90	481	84	0.7	7.9	4.3	232	81	250	188	62	108	5.7	128	83	1	3	2	3	6
NASH	54	F	23.5	105	126	249	108	1.0	7.9	4.2	166	269	188	113	75	127	7.5	187	153	3	3	2	3	8

For the 26 patients, the two patients diagnosed with NAFL are listed from the top, followed by the 24 diagnosed as NASH, listed in ascending order of NAS and then ascending ALT value.

### FcγRIIb distribution

**Fig 1:**
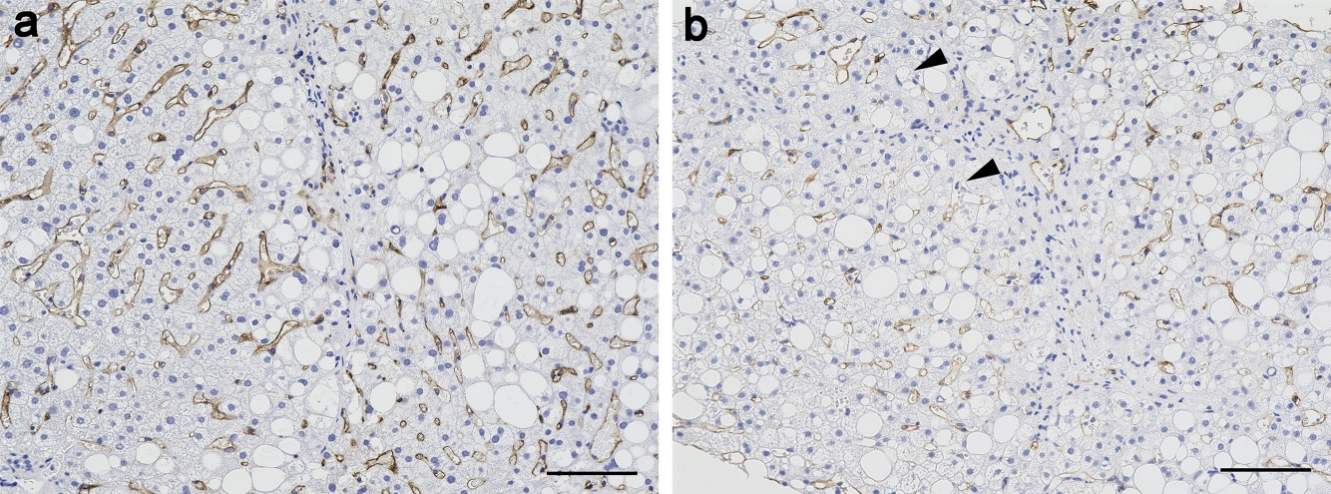
FcγRIIb expression and localization in liver tissues of patients. Representative light microscopic images of patients who were diagnosed as NASH (a: NAS, 2; b: NAS, 8). FcγRIIb signals detected with DAB (brown) are distributed in LSECs. In b, arrowheads indicate LSECs that do not detect FcγRIIb signals. Nuclei were counterstained with hematoxylin (blue). Scale bars represent 100 μm.

Representative light microscopic images of patients who were diagnosed as NASH (a: NAS, 2; b: NAS, 8) are shown in Fig 1. As it is well known, the FcγRIIb signals were found only in LSECs [25]. In a case with low NAS (= 2), FcγRIIb signals were strong and surrounded the entire circumference of the sinusoids (Fig 1A), whereas in a case with the highest NAS (= 8) among the cases we analyzed, FcγRIIb signals were very weak and were only irregularly expressed in LSECs (Fig 1B).

### FcγRIIb expression levels

For morphometric analysis, the FcγRIIb expression level was calculated as the ratio of the positive area to whole tissue area, using the morphometric procedure described in the materials and methods section (S1 Table). We also analyzed the FcγRIIb expression level as the intensity of positive signals (S1 Table), and this showed a similar tendency to the measurement results stated above. Hereinafter, the ratio of the FcγRIIb positive area is denoted as the FcγRIIb expression level in this paper.

### Correlation with biological indexes and serum biochemical markers

**Fig 2:**
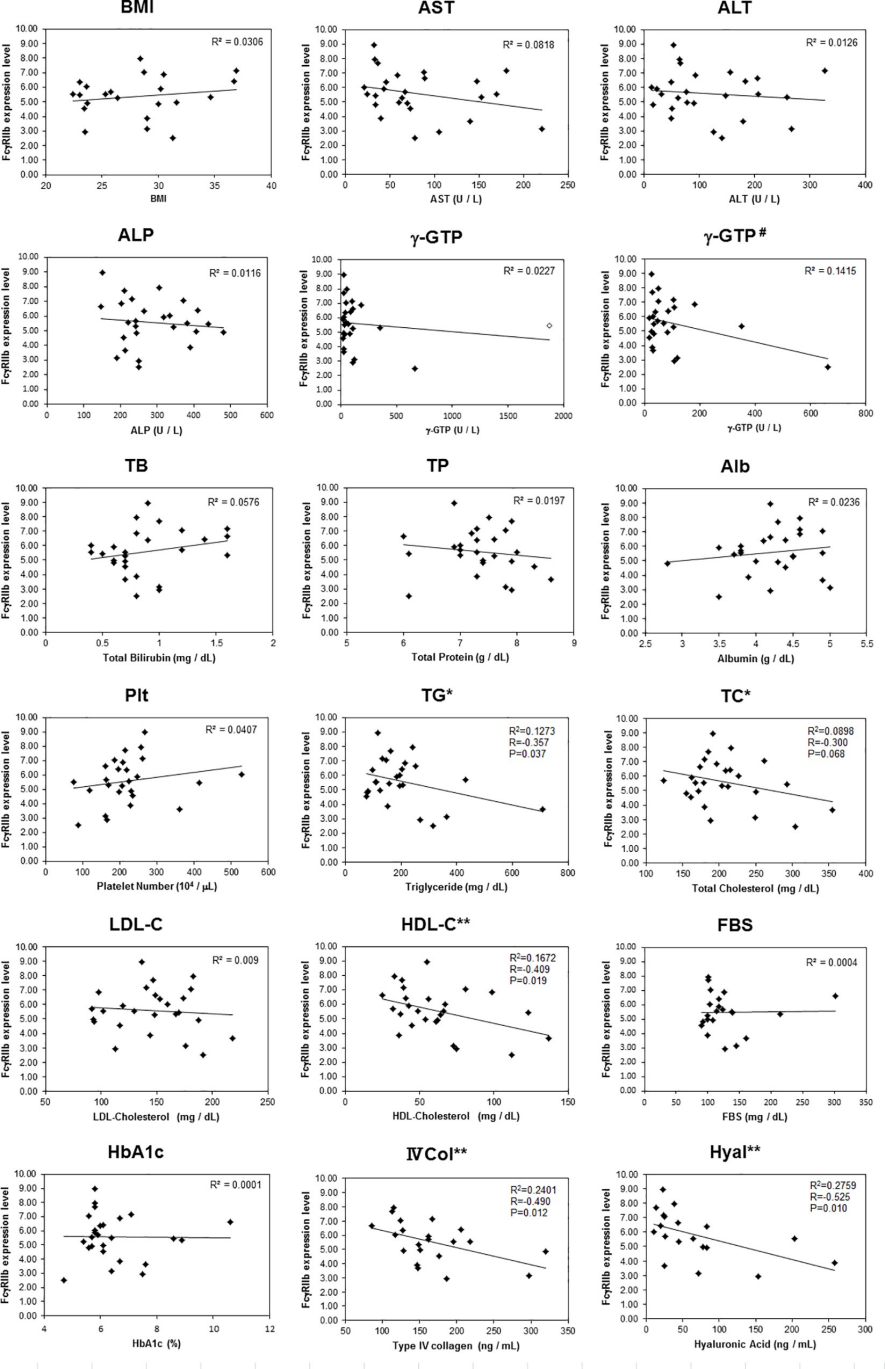
Correlation with biological indexes and biochemical data. Scatter plots show the correlation of FcγRIIb expression levels with BMI, Plt, and serum biochemical data. Statistical correlation was analyzed by the Pearson correlation coefficient test. #: one outlier (indicated as ◇ in γ-GTP diagram) removed. *: small correlation by the Pearson correlation coefficient test (R<-0.3). **: medium correlation by the Pearson correlation coefficient test (R<-0.4).

The correlations of FcγRIIb expression levels with BMI, Plt, and serum biochemical markers are shown in Fig 2. The Pearson correlation test indicated that there was no correlation between FcγRIIb levels and BMI. The regression lines of serum AST, ALT, ALP, and γ-GTP showed negative slopes, but there was no significant correlation. The regression lines showed positive slopes for TB, Alb, and Plt, which decreased with liver disease deterioration, though there was no significant correlation. On the other hand, in the factors related to lipid dynamics, it was shown that the FcγRIIb levels had a small negative correlation (R = -0.357, -0.300) with TG and TC, and a medium negative correlation (R = -0.409) with HDL-C. With LDL-C, there was no significant correlation, but the regression line also showed a negative slope, suggesting that it is similar to other lipid-related factors. In FBS and HbA1c, there was no correlation between FcγRIIb levels, meaning there was no correlation of FcγRIIb levels with short- and medium-term blood glucose levels. Both IVCol and hyaluronan, which increase with progression of hepatic fibrosis, showed medium negative correlation (R = - 0.490, -0.525).

### Comparisons of diagnosis, sex, and pathological scores

**Fig 3:**
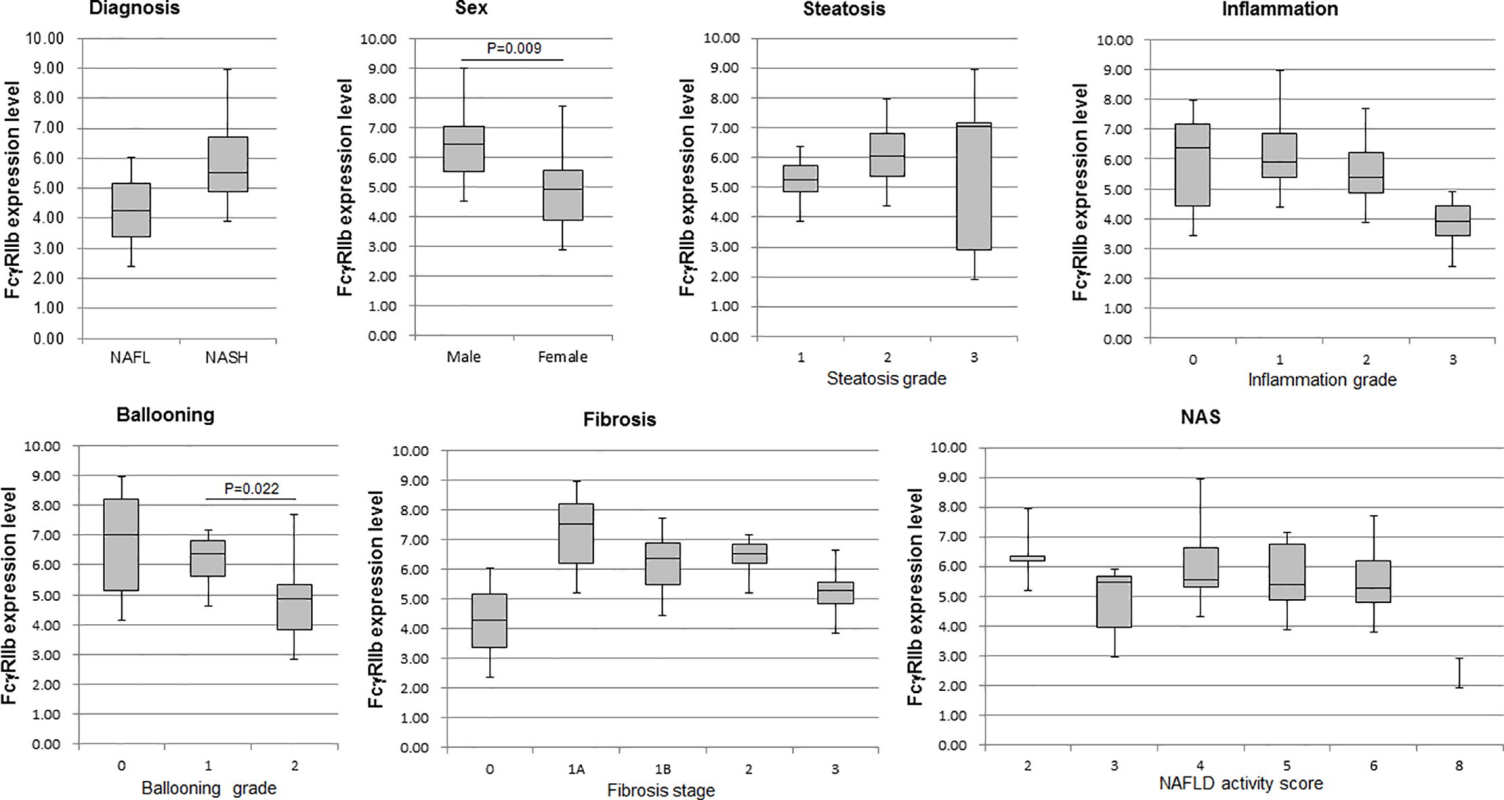
Comparisons of diagnosis, sex, and pathological scores. Box plots show FcγRIIb expression levels. Lines inside the boxes denote medians, the boxes represent the interquartile range, and whiskers extend to the minimum and maximum. The statistical differences among groups are analyzed by the Mann-Whitney or the Kruskal-Wallis test. A P-value <0.05 was considered to be significant.

The comparisons of diagnosis, sex, and pathological values are shown in Fig 3. FcγRIIb levels were significantly higher in females compared with in males (Mann-Whitney test, P = 0.009), whereas there was no significant difference in FcγRIIb expression levels of the liver biopsy specimen among the pathological grades of NAFLD. The mean FcγRIIb levels tended to be higher with the progression of steatosis, but no significant difference was observed. FcγRIIb levels became lower with the progression of inflammation and ballooning. Especially, it was significantly lower in patients with ballooning stage 3 as compared with those in stage 2 (Kruskal-Wallis test, P = 0.022). Interestingly, in the comparison between the fibrosis stages, which are closely related to the exacerbation of NASH, FcγRIIb levels tended to be higher at the early stage of fibrosis and became slightly lower at the 1B, 2 and 3 fibrosis stages. This is consistent with the findings that there were medium negative correlations between FcγRIIb levels and IVCol or hyaluronan. It is also suggested that the decline of FcγRIIb levels in the progression of NAFLD correlates with the exacerbation of fibrosis.

### Stabilin-2 distribution

High amounts of HDL-C, IVCol, and hyaluronan in peripheral blood coincided with low expression of FcγRIIb. Interestingly, some of these molecules are ligands for LSEC receptors other than FcγRIIb. Then, we examined the distribution of stabilin-2, a hyaluronan receptor in liver from patients with NASH. Under the microscopic observation, stabilin-2 positive LSECs tended to be more frequent in patients with low level of blood hyaluronan (S2 Fig), but nonspecific signals were detected in some biopsy specimen. Thus, we were unable to elucidate the expression levels of stabilin-2 during NASH.

## Discussion

In this study, we determined the expression levels of FcγRIIb, which is a scavenger receptor on the LSEC, in human NAFL and NASH biopsy specimens, and elucidated their correlations with individual biological indexes and pathological grades. The FcγRIIb expression levels indicated significant negative correlations with serum TG, TC, HDL-C, VICol, and hyaluronan concentrations, respectively.

Capillarization of LSECs is one of the basic pathological features of hepatic cirrhosis and chronic hepatitis [34]. LSECs of cirrhosis patients produce IVCol, fibronectin, and laminin, lose their characteristic fenestrations, and the expression levels of LSEC receptors LYVE-1, FcγRIIb and stabilin are decreased [9]. The microenvironment caused by the transformed LSECs is involved in the onset and progression of hepatic fibrosis, and instructively dictates hepatic regeneration and healing [35, 36]. In a rodent NASH model, capillarization arises at an early stage of NASH and exacerbates with NASH progression [11].

In human LSECs, the distribution pattern of FcγRIIb has recently been shown in intrahepatic lobules of normal livers [37]. In relation to hepatic diseases, the expression levels of LSEC markers, including FcγRIIb, of HCC patients were sequentially lost during tumor progression [22], and it disappeared in 63% of cases in the peritumoral tissue samples of a tissue microarray of HCC [7].

Interestingly, several of the serum components that showed negative correlations with the expression of FcγRIIb are ligands for LSEC receptors: hyaluronan, which is the first molecule shown to be endocytosed via stabilin in LSECs, HDL-C, which binds to a receptor in LSEC, and IVCol, which probably binds to the Mannose receptor in LSEC [17, 38].**　** These serum components are well known to increase with the progression of NASH. The increase of individual serum components indicates the progression of hepatic injury in the NASH patients and probably reflects the functional status of scavenger receptors expressed in LSECs. Our results indicate that the decrease of FcγRIIb expression may also be closely related to its scavenger function, involving several LSEC receptors.

Hyaluronic acid synthesized by mesenchymal cells is generally eliminated by LSECs. Serum hyaluronic acid contents increase in alcoholic liver disease, chronic hepatitis C, and NAFLD [39–41]. Recently, it was demonstrated that the index of hyaluronic acid in patients can accurately predict the survival rate of liver diseases of varying severity [20].　Here, we attempted to elucidate the expression of stabilin-2 proteins, one of the hyaluronan receptors, in liver biopsy tissue of NASH patients, but we were unable to elucidate its relationship with NASH progression. However, there are several reports indicating the relationship between stalibin-2 and FcγRIIb expression in HCC. Geraud and colleagues examined the expression of FcγRIIb and stabilin-2 by using a tissue microarray of HCC patients and revealed the loss of both receptor proteins in the majority of tumorous tissues [22]. In the peri-tumorous liver tissues, loss of stabilin-2 expression was significantly less likely to occur (38%) than loss of FcγRIIb expression (63%), and the loss of stabilin-2 in peri-tumorous tissues was associated with survival. They described that loss of stabilin-2 may enhance survival by preventing endothelial-tumor cell adhesive interactions and microvascular invasion. Schledzewski and colleagues revealed that serum hyaluronic acid levels were elevated in stabilin-1 and stabilin-2 double KO mice, but the liver showed only mild perisinusoidal fibrosis without dysfunction [42]. They also indicated that the expression levels of FcγRIIb and LYVE-1 in double KO mice were not different from WT mice. From these previous observations, the attenuation pattern of individual LSEC receptors appears to vary depending on the type of liver disease and the degree of progression. The attenuation of FcγRIIb in LSECs seems to be involved in inflammation and fibrosis in NASH patients, at the least. Platelets are also closely involved in inflammation and fibrosis, and the platelet count of NASH patients decreases with progressive fibrosis [43]. Platelets mediate binding of endothelial cells to T cells during hepatic injury [44], and promote various processes such as inflammation, fibrosis and liver repair by release of biological substances [45]. In this study, although there was no correlation between platelet count and FcγRIIb expression levels, the regression line indicates a positive slope. In other words, patients with low FcγRIIb expression levels exhibit a tendency to have a low platelet count (Fig 2), a high inflammation grade, or a high fibrosis stage (Fig 3). FcγRIIb expression levels may be related to the enhancement of inflammation and fibrosis in NASH patients.

The phenotypic changes of LSECs associated with various liver diseases have been clarified in detail. In the LSEC studies, contradictory results may be shown depending on species differences, phenotypic markers used for identification, and culture conditions [46]. Here, we showed that FcγRIIb expression may coincide with scavenger functions of several LSEC ligands related to inflammation, hepatic fibrosis, and hepatic lipid metabolism. These findings are useful to clarify details of transformation and dysfunction in LSECs during NAFLD. Further investigations are required to assess the significance and to address the role of scavenger receptors, including FcγRIIb, in LSECs during NASH progression.

## Conclusions

In summary, the present study contributes to support the notion that the changes in LSEC phenotype are one of crucial events in human NASH progression. Our results suggest that progression of inflammation, hepatic fibrosis, and hepatic lipid metabolism disorder during NASH may influence the expression and scavenger functions of FcγRIIb in LSECs.

## Supporting information

S1 Fig Morphometric imaging for determining FcγRIIb expression levels. Representative images of patients who were diagnosed as NASH (a: NAS, 2; b: NAS, 8). Whole tissue area (other than the area shown in black) and FcγRIIb expression area (pink) were automatically extracted under a determined procedure. In b, arrowheads indicate LSECs that do not detect FcγRIIb signals. FcγRIIb signals detected in LSECs were accurately extracted. Scale bars represent 100 μm.
(TIF)

S2 Fig Stabilin-2 detection in NASH. Representative light microscopic images of liver tissues in NASH patients with (a) blood hyaluronan: 23 ng/ml; FcgRIIb level: 8.97%; fibrosis stage: 1A; NAS: 4, (b) blood hyaluronan: 258 ng/ml; FcgRIIb level: 3.87%; fibrosis stage: 3; NAS: 5. Immunohistochmical signals of stabilin-2 in LSECs are detected (arrowheads). Unfortunately, nonspecific signals are also detected in b. Nuclei were counterstained with hematoxylin (blue). Scale bars represent 100 μm.
(TIF)

S1 Table FcgR2b expression level. For the 26 patients, the two patients diagnosed with NAFL are listed from the top, followed by the 24 diagnosed as NASH, listed in ascending order of NAS and then ascending ALT value. The order of patients in this table is the same as Table 1. A comparison with the Fibrosis score and FcgR2b expression level is stated in Discussion. * FcgR2b positive area / whole tissue area.
** Intensity of FcgR2b positive area / whole tissue area.
(XLSX)
