# Supplementary figures and images for: Fc gamma RIIb expression levels in human liver sinusoidal endothelial cells during progression of non-alcoholic fatty liver disease
Source: PLoS One. 2019 Jan 29;14(1):e0211543. doi: 10.1371/journal.pone.0211543 (PMC6350999; doi:10.1371/journal.pone.0211543)

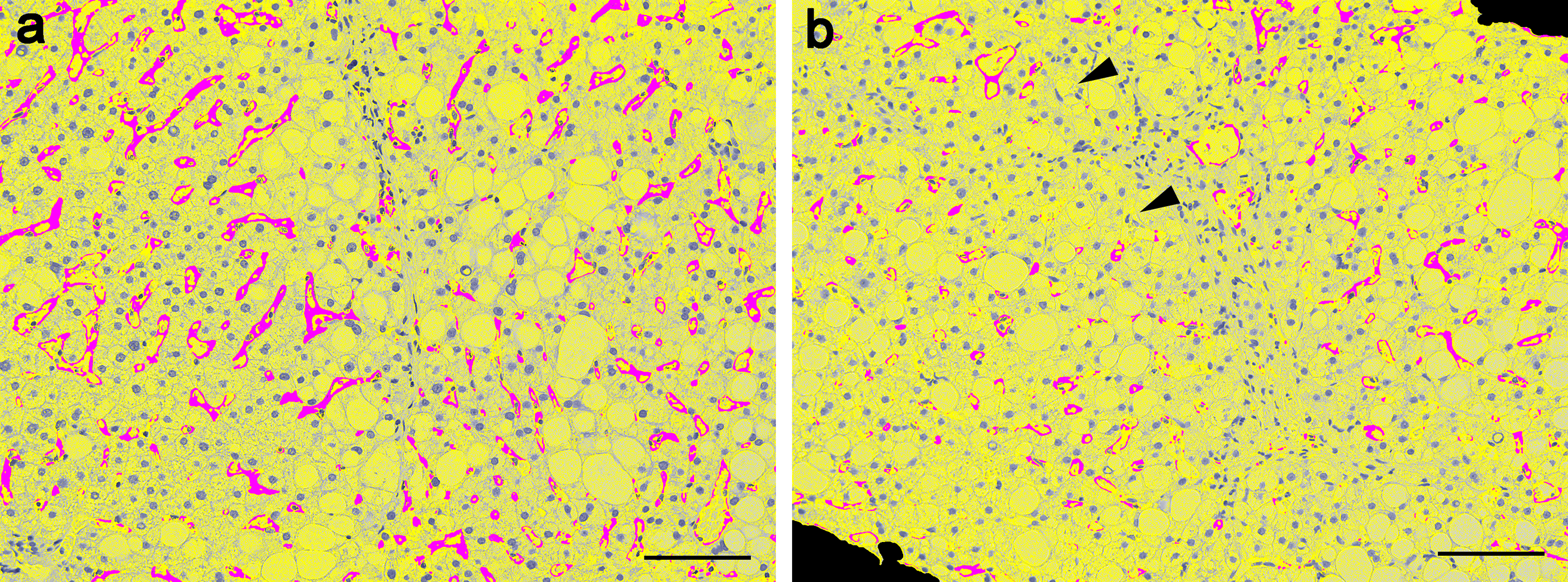

Supplement: S1 Fig — Representative images of patients who were diagnosed as NASH (a: NAS, 2; b: NAS, 8). Whole tissue area (other than the area shown in black) and FcγRIIb expression area (pink) were automatically extracted under a determined procedure. In b, arrowheads indicate LSECs that do not detect FcγRIIb signals. FcγRIIb signals detected in LSECs were accurately extracted. Scale bars represent 100 μm. (TIF) [file pone.0211543.s001.tif]

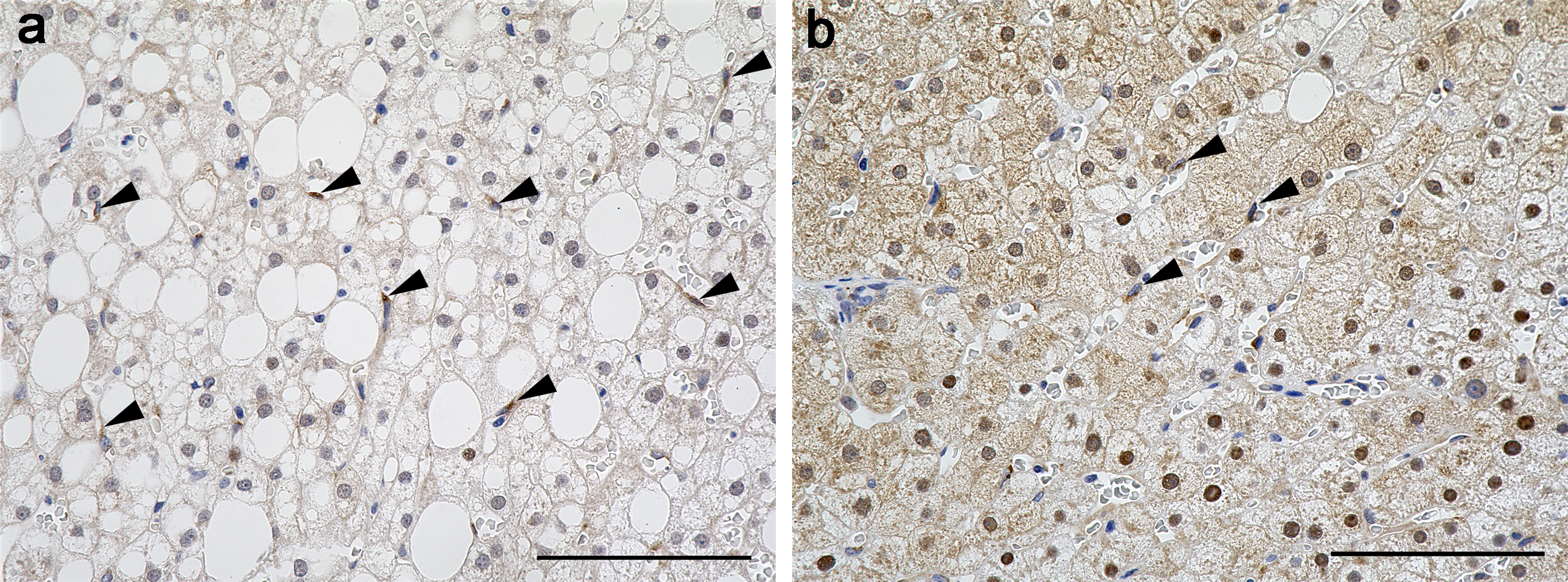

Supplement: S2 Fig — Representative light microscopic images of liver tissues in NASH patients with (a) blood hyaluronan: 23 ng/ml; FcgRIIb level: 8.97%; fibrosis stage: 1A; NAS: 4, (b) blood hyaluronan: 258 ng/ml; FcgRIIb level: 3.87%; fibrosis stage: 3; NAS: 5. Immunohistochmical signals of stabilin-2 in LSECs are detected (arrowheads). Unfortunately, nonspecific signals are also detected in b. Nuclei were counterstained with hematoxylin (blue). Scale bars represent 100 μm. (TIF) [file pone.0211543.s002.tif]
